# Successful negotiation of goal conflict between romantic partners predicts better goal outcomes during COVID-19: A mixed methods study

**DOI:** 10.1177/02654075211033341

**Published:** 2022-02

**Authors:** Laura M. Vowels, Katherine B. Carnelley, Rachel R. R. Francois-Walcott

**Affiliations:** University of Southampton, UK

**Keywords:** COVID-19, goals, goal conflict, interpersonal relationships, mixed methods

## Abstract

When romantic partners’ personal goals conflict, this can negatively affect personal goal outcomes, such as progress. In a concurrent mixed methods study, we investigated whether goal conflict and negation of goal conflict were associated with goal outcomes (progress, confidence, motivation) and what strategies partners used during the COVID-19 pandemic to negotiate goal conflict. Survey participants (*n* = 200) completed a daily diary for a week and weekly longitudinal reports for a month and interview participants (*n* = 48) attended a semi-structured interview. Results showed that higher goal conflict was associated with lower goal outcomes, and successful negotiation of goal conflict was associated with better goal outcomes. Qualitative analyses identified three goal conflict negotiation strategies (compromise, integration, concession). Conversations focused on both practical and emotional needs and included respectful communication and space from conflict (timeout or avoidance). The mixed methods results suggest that goal conflict was low during the pandemic and participants were often able to negotiate goal conflict resulting in better goal outcomes.

Close relationship partners play an important role in each other’s pursuit of personal goals (Cappuzzello & Gere, 2018; Feeney & Collins, 2015; Overall et al., 2010). Individuals in relationships spend a great deal of time together and become increasingly interdependent over time leading to a greater influence over each other’s behavior (Rusbult & Van Lange, 2003). Previous research has shown that individuals in supportive relationships make more progress toward their goals and experience increased individual and relational well-being (Drigotas et al., 1999; Feeney, 2004; Tomlinson et al., 2016). However, when partners’ goals conflict, maintaining goals may be difficult leading to worse goal outcomes (i.e., making less progress, feeling less committed toward goals, and feeling less confident about being able to achieve their goals). On the other hand, if partners successfully negotiate instances of goal conflict, this may lead to better goal outcomes.

In the present study, we employed a concurrent mixed methods design in which both quantitative and qualitative data were collected simultaneously to complement each other. In the quantitative component, our aim was to add to the extant literature by examining whether low goal conflict and successful negotiation of goal conflict were associated with better goal outcomes across multiple time-points. The qualitative data described how couples negotiate instances of goal conflict to better understand how couples may be able to minimize the potential negative impact of goal conflict. Because the data were collected during the COVID-19 pandemic, we also examined whether goal conflict and negotiation of goal conflict predicted perception of the pandemic affecting participants’ goals and asked participants in the qualitative interviews how they negotiated goal conflict during the pandemic.

## Interdependence theory, goal conflict, and personal goal outcomes

Interdependence theory (Kelley & Thibaut, 1978; Rusbult & Van Lange, 2003) proposes that relationship partners become increasingly interdependent over time as the relationship progresses and couples spend an increasing amount of time interacting together. Each partner’s needs, thoughts, and motives will influence interactions with one another and depending on the congruence between the partners, interactions can be perceived positively or negatively (Rusbult & Van Lange, 2003). Increasing interdependence means that partners begin to influence each other’s decision-making and each partner’s actions have implications for the other partner. Thus, interdependent romantic relationship partners need to learn to coordinate goal-directed activities. During the COVID-19 pandemic, partners who live together are likely to become increasingly interdependent over the course of the pandemic as most will spend more time together and have fewer outside resources available to them. At the same time, partners are likely to experience goal conflict as they are having to negotiate how to manage the new circumstances including childcare, working from home, and potentially one or both partners being off work.

Repeated exposure to goal conflict is likely to be harmful for relationships because it continuously tests partners’ commitment toward each other (Kelley & Thibaut, 1978; Rusbult & Van Lange, 2003). In fact, several studies have shown goal conflict to be negatively associated with relationship quality as well as personal well-being (Gere & Impett, 2018; Gere & Schimmack, 2013; Gere et al., 2011; Righetti et al., 2016). Another study has shown that when people find it difficult to sacrifice or make a change for their partner or the relationship, they feel less satisfied in the relationship (Ruppel & Curran, 2012). People are also less motivated to provide support toward their partner’s goals when they feel goals might take their partner away from the relationship (Feeney et al., 2013, 2017), especially if they are highly invested in the relationship (Hui et al., 2014). It is evident from the research that there are a number of potential costs associated with goal conflict including loss of support and lowered well-being.

Therefore, partners may be motivated to reduce instances of goal conflict to reduce stress on the system. Indeed, previous research has found that a high level of goal conflict in a relationship makes it more difficult to coordinate goal pursuit and individuals are less likely to make progress toward goals that are problematic for the relationship (Gere & Schimmack, 2013). A recent study showed that individuals were more likely to stop pursuing or devalue a goal if it conflicted with their partner’s goals (Gere & Impett, 2018). Over time, devaluing goals that were conflicting predicted greater commitment toward the relationship partner (Gere & Impett, 2018). Previous research has assessed the impact of goal conflict on goal outcomes only cross-sectionally or across two time points. The present study adds to the literature by assessing the association between goal conflict and goal outcomes in a longitudinal dataset collected over a number of days and weeks.

Furthermore, being able to successfully negotiate goal conflict is likely to predict better goal outcomes. Indeed, the transactive goal dynamics theory, which is based on interdependence theory, proposes that romantic partners become an interdependent system which regulates goals for both individuals as well as the relationship (Fitzsimons et al., 2015). The theory suggests that when partners can agree on which goals to pursue, how to pursue them, and how independently, they will be more successful in negotiating goal pursuit and experience better goal outcomes for both personal and relational goals. However, we are aware of no studies to date that have directly assessed how negotiation of conflict, or goal coordination, in goal situations predicts goal outcomes. The present study aims to add to the literature by testing the assumption of the transactive goal dynamics theory (Fitzsimons et al., 2015) that successful goal coordination predicts better goal outcomes.

Finally, in addition to examining whether successful negotiation would predict better goal outcomes, we also wanted to understand what successful negotiation of goal conflict looked like for people. We are aware of no studies to date that have attempted to categorize context-specific negotiation strategies for goal conflict specifically. Therefore, we can examine the literature on accommodation to understand what types of strategies partners might use to negotiate goal conflict during the pandemic. Research into accommodation has shown that more constructive reactions (e.g., actively talking about the problem [voice] or passively prioritizing or sacrificing for the relationship [loyalty]) to potential conflict predicts better relationship satisfaction, commitment, and overall happiness compared to destructive strategies (e.g., actively picking a fight [exit] or passively deciding the partner cannot be trusted anymore [neglect]; Rusbult et al., 1982). The present study adds to the literature by providing a qualitative analysis of which strategies individuals use to negotiate conflict.

## The current study

We used mixed methods to test our research questions and hypotheses; quantitative data can answer questions more broadly and generally and qualitative data provides more nuanced and detailed insights into how the pandemic has impacted participants’ ability to negotiate goal conflict. We expected that when an individual’s goal conflicts with the partner’s or relationship’s goals, they will report lower goal outcomes (progress, confidence, motivation; H1). We expected that successful negotiation of goal conflict will be positively associated with goal outcomes (a novel hypothesis; H2). To understand which strategies participants in the study may have used, we conducted semi-structured interviews and asked participants to describe how they negotiated any potential goal conflict within their relationship (RQ1). We sampled both quantitative and qualitative participants over time to examine whether there were any changes in goal outcomes during the early pandemic. In the quantitative component, participants responded to questions once a day for a week and once a week for 5 weeks. This study is among the first to provide a window into how couples negotiate goals while living under stay-at-home orders due to COVID-19.

## Method

### Participants and procedure

We preregistered the study on the Open Science Framework (https://osf.io/6ebyz); data, code, and materials can be found here: https://osf.io/qr7cm. The study received ethical approval from the authors’ institutional review board. The quantitative data was collected via Prolific and social media was used to recruit participants for the semi-structured qualitative interviews. In order to be eligible for the study, participants had to be 18 years or over and living with their romantic partner in a country where social distancing measures were in place. We recruited 200 participants for the quantitative portion of the study. Based on a simulated power analysis, data from 200 participants (up to 4,200 observations) yield a power of 96.7% to estimate an average effect size in Psychology (r = 0.22, d = 0.45; Richard et al., 2003) with an alpha level of *p* < .01. Participants recruited through Prolific received £4.70 only if they completed all daily diary entries and another £2 if they completed all three additional follow-ups. If participants did not finish the daily diary, they were not compensated. Qualitative interview participants were entered into a raffle to win one of two £30 Amazon vouchers after the first interview and one of two £20 Amazon vouchers after the second interview.

Participants in the quantitative component completed a baseline survey on 31 March, 2020, shortly after many countries had gone under lockdown. The participants then completed a daily dairy survey over the next 7 days with the first entry completed directly after the baseline survey. After completing the daily diary, participants completed further three follow-up surveys that were each 1 week apart. This resulted in a total of 5 weekly time-points. Participants responded to questions regarding goal conflict, negotiation of goal conflict, and goal outcomes from the previous 24 hours in the daily diaries and from the previous week in the follow-up surveys. All surveys were conducted via Qualtrics. The final sample in the quantitative surveys was 200 with an attrition rate of 4% at the end of the daily diary and 8.5% at the end of the 5 weeks. However, all participants completed at least two time-points and were therefore included in the final analyses.

The semi-structured qualitative interviews were conducted via Zoom, audio recorded, and transcribed. Participants were asked questions about how they and their partner have negotiated instances of goal conflict during the pandemic. The first set of interviews were completed between 30 March 2020 and 21 April 2020. Participants recruited through Prolific were also eligible to participate in the interview. A total of 48 participants completed the first qualitative interview (30 were recruited via social media, 18 via Prolific). Participants who had completed the first interview in the first 2 weeks^1^ of the qualitative data collection were invited to participate in the follow-up interview a month later to better understand how support had changed over the course of the lockdown. Nineteen of the 23 participants invited to complete a second interview responded. Initial interviews lasted between 14 and 49 minutes and second interviews between 7 and 24 minutes.

Participants in quantitative and qualitative components of the study had similar demographic characteristics (see Table 1). Participants were on average 36 years old and had been in a relationship for 11 years. The samples were primarily white, heterosexual, and from the United Kingdom. Around half the participants were married and half cohabiting, and half of them had children. Only a small number of participants were keyworkers or had shown coronavirus symptoms. None had been diagnosed with coronavirus at baseline.

**Table 1.** Demographic variables.

	Quantitative (n = 200)		Qualitative (n = 48)	
	M	SD	m	SD
Age	36.5	12.3	36.0	12.9
Relationship length	11.1	9.32	10.4	10.9
	n	%	n	%
Gender				
Woman	105	52.5	33	68.8
Man	93	46.5	15	31.1
Other	2	1.0	0	0.0
Sexual orientation				
Heterosexual	182	91.0	36	76.6
Bisexual	9	4.5	7	14.9
Lesbian/Gay	7	3.5	4	8.5
Other	2	1.0	0	0.0
Relationship status				
Married	102	51.0	26	55.2
Cohabiting	98	49.0	22	46.8
Children				
No	95	47.5	33	70.2
Yes	105	52.5	13	29.8
Ethnicity				
White	184	92.0	41	87.2
Black	5	2.5	1	2.1
Asian	6	3.0	4	8.5
Mixed	2	1.0	1	2.1
Education				
Graduated high school	28	14.0	4	8.5
Some college	38	19.0	4	8.5
Undergraduate	74	37.0	17	36.1
Postgraduate	52	26.0	19	40.4
Other	8	4.0	4	8.5
Employment status				
Employed full-time	121	60.5	21	44.7
Employed part-time	23	11.5	6	12.8
Self-employed	26	13.0	6	12.8
Student	4	2.0	6	12.8
Unemployed	7	3.5	4	8.5
Retired	9	4.5	3	6.4
Employment changed				
No	153	76.5	33	70.2
Yes	47	23.5	14	29.8
Usually work from home				
No	138	69.0	33	70.2
Yes	62	31.0	13	27.7
Country				
UK	119	59.5	32	68.1
USA	17	8.5	4	8.5
Other	64	32.0	12	25.5
Keyworker				
No	166	83.0	44	93.6
Yes	34	17.0	3	6.4
Coronavirus symptoms				
No	179	89.5	39	83.0
Yes	21	10.5	8	17.0

### Measures

At each time-point, participants listed up to three goals (these could be any goals) that they had been working toward in the past 24 hours (or the past week in the weekly follow-ups). Participants reported the following types of goals: domestic (31.4%), exercise/health (20.1%), career (16.4%), hobbies/self-development (14.7%), relationships (6.3%), self-care (4.2%), education (2.8%), COVID-related (2.8%), and finance (1.3%).

#### Goal outcomes

Participants then answered a set of questions for each goal using 1 item for each: “How much progress did you actually make toward achieving this goal?” (progress); “How motivated did you feel in working toward this goal?” (motivation); and “How confident did you feel in being able to achieve this goal?” (confidence). Participants were also asked how much they felt the pandemic had affected their goal pursuit overall (affected).

#### Goal conflict and negotiation

Goal conflict was measured with 2 items, one for conflict with partner’s goals and one for relationship’s goals: “How problematic was pursuing this goal for your partner/relationship?” (conflict; *r* = .80). Participants were also asked “How well were you able to negotiate with your partner being able to work toward your goals?” (negotiate) All items were rated on a scale from 0 (*Not at All*) to 10 (*Extremely*), except goal progress which was rated on a scale from 0 to 100%.

#### Qualitative semi-structured interviews

We asked participants a range of questions about their relationship and goal pursuit during the pandemic. The questions relevant for this report were “How have you negotiated any goal conflicts between you and your partner when you have tried to work toward tasks and goals?” and “Are there any specific strategies that you found helpful or haven’t worked?”

### Quantitative analysis plan

It is important to understand whether the results are driven by within- or between-participants factors. Therefore, we separated the within- and between-subjects’ elements of the predictor variables (see Bolger &Laurenceau, 2013 for more details). The within-subjects variables show the difference in the outcome variables due to within-person change day-to-day and the between-subjects variables show the average difference between participants in the outcome variables. Time was scaled to start at 0 and was included in both daily diary (days 0–6) and weekly analyses (days 0–27). Daily diary data and the weekly longitudinal data were both separately analyzed using hierarchical linear modeling with restricted maximum likelihood estimation (REML) to account for missing data (Raudenbush & Bryk, 2002). All participants were measured on the same days and therefore we did not include random variability at the day/week level. Goal conflict and goal outcomes were measured 3 times for each time-point, once for each goal, and therefore the analyses included three levels with two levels of random variability. Negotiation of goal conflict and the effect of coronavirus pandemic on goals were only measured once at each time-point and therefore only included two levels. We only included a random intercept in the models as models with random slopes failed to converge. All quantitative data were analyzed using the *lme4* package in *R*. We used an alpha level of *p* < .01 as a cutoff for significance to account for multiple analyses. Descriptive statistics and zero-order correlations among all study variables are presented in Table 2.

**Table 2.** Means, standard deviations, and correlations with confidence intervals.

Variable	*M*	*SD*	1	2	3	4	5	6
1. Conflict	1.36	2.14	—	−.20**	−.13**	−.13**	−.06*	.05
				[−.24, −.16]	[−.17, −.09]	[−.16, −.09]	[−.09, −.02]	[.01, .09]
2. Negotiate	7.14	2.60	−.16**	—	.13**	.10**	.09**	−.02
			[−.19, −.13]		[.09, .17]	[.06, .14]	[.05, .13]	[−.06, .01]
3. Progress	68.64	30.87	−.10**	.14**	—	.52**	.41**	−.04
			[−.13, −.07]	[.11, .17]		[.49, .55]	[.37, .44]	[−.09, −.01
4. Confidence	7.02	2.55	−.12**	.11**	.53**	—	.60**	−.01
			[−.15, −.09]	[.07, .14]	[.51, .56]		[.57, .62]	[−.05, .03]
5. Motivation	7.00	2.61	−.05*	.12**	.45**	.59**	—	.03
			[−.09, −.02]	[.08, .15]	[.43, .48]	[.56, .61]		[−.02, .06]
6. Affected^a^	4.82	3.25	.07**	−.02	−.07**	−.06**	−.03	—
			[.03, .10]	[−.05, .02]	[−.10, −.03]	[−.09, −.03]	[−.06, −.00]	

*Note. M* and *SD* are used to represent mean and standard deviation, respectively. Values in square brackets indicate the 95% confidence interval for each correlation. The correlation in the daily diary data are presented below the diagonal and weekly measures above the diagonal.* indicates *p* < .01. ** indicates *p* < .001. P = conflict with partner’s goals, R = conflict with relationship’s goals.

^a^ Perception of goals being affected by the pandemic.

### Qualitative analysis plan

We analyzed the qualitative interviews using codebook thematic analysis (Braun & Clarke, 2006, 2019) with NVivo 12.0 software. We used a combination of inductive and deductive approaches to coding by using previous theory and research to guide coding but allowing new codes to be created throughout the coding process. The first and third author coded the interviews and both familiarized themselves with the data before creating the initial low-level codes. Codes were created by coding each meaning unit which may have been one word, sentence, or paragraph. These codes were then refined iteratively by the two coders and the final themes were agreed jointly. Any disagreements were discussed until 100% agreement was reached on the coding. “[…]” was used in the quotes if unnecessary detail was removed or to provide needed additional information in the quoted data. Repeated filler words such as “like” and “yeah” were excluded to aid readability. Identifying information was removed.

### Mixed methods

We used a concurrent mixed methods design in which both quantitative and qualitative data were collected simultaneously: The quantitative data provided information on how goal conflict and negotiation of goal conflict were associated with a range of goal outcomes, whereas the qualitative results provided more nuanced information on what types of strategies participants employed to successfully negotiate instances of goal conflict. The present research was fundamentally guided by pragmatism in line with mixed methods research: the research questions were seen as the primary importance regardless of the philosophical worldview or the method (Creswell & Plano Clark, 2007). Quantitative research is often seen as positivist or postpositivist which can be at odds with qualitative research as it is inherently more interpretive in nature (Lincoln et al., 2011). Given the unprecedented nature of the pandemic, we believe that using a combination of methods enabled us to gain a more thorough understanding of partner support during the pandemic than using any one method alone could have accomplished. The quantitative and qualitative results are combined to describe the overall functioning of individuals in relationships during COVID-19.

## Results

### Quantitative results

#### Goal conflict and goal outcomes

We hypothesized that greater perceived goal conflict would be associated with lower goal outcomes during the pandemic (H1; see Table 3 for results^2^). In line with the hypothesis, on days/weeks when participants perceived higher levels of goal conflict, they also reported less goal progress, confidence, and motivation compared to days when they perceived lower levels of goal conflict.^3^ The results also showed that on average, participants who experienced higher levels of goal conflict reported lower levels of goal progress and confidence but not motivation compared to participants who reported lower levels of goal conflict (between-participant change).

**Table 3.** Results from the hierarchical linear modeling for goal conflict as a predictor of goal outcomes.

	Progress						Confidence						Motivation					
	Daily			Weekly			Daily			Weekly			Daily			Weekly		
*Predictors*	*Estimates*	*CI*	*p*	*Estimates*	*CI*	*p*	*Estimates*	*CI*	*p*	*Estimates*	*CI*	*p*	*Estimates*	*CI*	*p*	*Estimates*	*CI*	*p*
Intercept	65.47	62.69 to 68.25	**<0.001**	66.71	63.92 to 69.50	**<0.001**	7.16	6.94 to 7.38	**<0.001**	7.18	6.95 to 7.42	**<0.001**	7.08	6.85 to 7.31	**<0.001**	7.10	6.85 to 7.34	**<0.001**
Conflict_W_	−1.62	−2.16 to −1.08	**<0.001**	−2.18	−2.85 to −1.50	**<0.001**	−0.16	−0.21 to −0.12	**<0.001**	−0.18	−0.23 to −0.12	**<0.001**	−0.07	−0.12 to −0.03	**0.002**	−0.08	−0.14 to −0.02	**0.006**
Conflict_B_	−2.35	−4.00 to −0.71	**0.005**	−2.80	−4.37 to −1.24	**<0.001**	−0.20	−0.33 to −0.07	**0.003**	−0.24	−0.37 to −0.11	**<0.001**	−0.15	−0.28 to −0.01	0.032	−0.17	−0.30 to −0.03	0.017
Time	0.50	0.07 to 0.93	0.021	0.12	0.02 to 0.22	0.018	−0.03	−0.06 to 0.01	0.123	−0.01	−0.02 to −0.00	**0.002**	−0.01	−0.05 to 0.03	0.583	−0.01	−0.02 to 0.00	0.070
**Random Effects**																		
σ^2^	709.36			659.79			4.68			4.29				5.00		4.69		
τ_00_	280.87 _ID_			261.77 _ID_			1.76 _ID_			1.95 _ID_				1.87 _ID_		2.03 _ID_		
ICC	0.28			0.28			0.27			0.31				0.27		0.30		
N	200 _ID_			199 _ID_			200 _ID_			199 _ID_				200 _ID_		199 _ID_		
Observations	3755			2660			3773			2676				3769		2673		
R^2^	0.021			0.033			0.025			0.035				0.010		0.013		

*Note.* W = within-participant, B = between-participant

Significant associations are denoted in bold in the table.

In addition to goal conflict, we hypothesized that the perception of how well participants had been able to negotiate goal conflict predicted goal outcomes during the pandemic (H2; see Table 4 for results). We found that on days/weeks when participants reported more successful negotiation of goal conflict, they reported experiencing better goal outcomes compared to days/weeks with less successful negotiation of goal conflict. The results showed a similar pattern for between-participants: participants who reported more successful negotiation of goal conflict overall also reported better goal outcomes on average compared to participants who reported higher levels of goal conflict.

**Table 4.** Results from the hierarchical linear modeling for negotiation of goal conflict as a predictor of goal outcomes.

	Progress						Confidence						Motivation					
	Daily			Weekly			Daily			Weekly			Daily			Weekly		
*Predictors*	*Estimates*	*CI*	*p*	*Estimates*	*CI*	*p*	*Estimates*	*CI*	*p*	*Estimates*	*CI*	*p*	*Estimates*	*CI*	*p*	*Estimates*	*CI*	*p*
Intercept	65.22	62.57 to 67.88	**<0.001**	66.55	63.91 to 69.18	**<0.001**	7.13	6.93 to 7.33	**<0.001**	7.18	6.97 to 7.38	**<0.001**	7.06	6.86 to 7.26	**<0.001**	7.09	6.88 to 7.30	**<0.001**
Negotiate_W_	2.16	1.65 to 2.68	**<0.001**	2.11	1.46 to 2.75	**<0.001**	0.13	0.09 to 0.18	**<0.001**	0.13	0.08 to 0.18	**<0.001**	0.15	0.11 to 0.20	**<0.001**	0.12	0.07 to 0.18	**<0.001**
Negotiate_B_	3.46	2.32 to 4.61	**<0.001**	3.93	2.81 to 5.05	**<0.001**	0.39	0.30 to 0.47	**<0.001**	0.45	0.37 to 0.54	**<0.001**	0.41	0.33 to 0.49	**<0.001**	0.45	0.37 to 0.54	**<0.001**
Time	0.54	0.11 to 0.97	0.013	0.13	0.02 to 0.23	0.016	−0.02	−0.06 to 0.01	0.214	−0.01	−0.02 to −0.00	**0.002**	−0.01	−0.04 to 0.03	0.697	−0.01	−0.02 to 0.00	0.076
**Random Effects**																		
σ^2^	703.05			659.18			4.70			4.33			4.96			4.67		
τ_00_	246.70 _ID_			218.16 _ID_			1.24 _ID_			1.23 _ID_			1.22 _ID_			1.23 _ID_		
ICC	0.26			0.25			0.21			0.22			0.20			0.21		
N	200 _ID_			199 _ID_			200 _ID_			199 _ID_			200 _ID_			199 _ID_		
Observations	3738			2648			3756			2664			3752			2661		
R^2^	0.064			0.082			0.103			0.145			0.111			0.135		

*Note.* W = within-participant, B = between-participant, Significant associations are denoted in bold in the table.

Although not preregistered, we also explored whether goal conflict and negotiation of goal conflict were associated with a perception that the pandemic was affecting goal pursuit (see Table 5 for results). We found that on days/weeks when goal conflict was higher, participants reported that their goals were affected by the pandemic more than on days/weeks when goal conflict was lower. Similarly, at the between-participant level, participants who reported higher levels of goal conflict overall also reported that the pandemic was having more of an impact on their goal pursuit compared to participants who reported lower levels of goal conflict. In contrast, negotiation of goal conflict was not associated with participants’ perception of their goals being affected by the pandemic.

**Table 5.** Results from the hierarchical linear modeling for goal conflict and negotiation of goal conflict as predictors of participants’ perception of goals being affected by the pandemic in separate models.

	Goals Being Affected by the Pandemic					
	Daily			Weekly		
*Predictors (Goal Conflict)*	*Estimates*	*CI*	*p*	*Estimates*	*CI*	*p*
Intercept	5.05	4.71 to 5.40	**<0.001**	4.83	4.48 to 5.18	**<0.001**
Conflict_W_	0.09	0.05 to 0.14	**<0.001**	0.07	0.02 to 0.12	0.011
Conflict_B_	0.59	0.38 to 0.81	**<0.001**	0.53	0.32 to 0.75	**<0.001**
Time	−0.13	−0.16 to −0.09	**<0.001**	−0.00	−0.01 to 0.00	0.378
**Random Effects**						
σ^2^	5.40			4.42		
τ_00_	5.23 _ID_			5.54 _ID_		
ICC	0.49			0.56		
N	200 _ID_			199 _ID_		
Observations	4080			2844		
R^2^	0.075			0.063		
*Predictors (Negotiation)*	*Estimates*	*CI*	*p*	*Estimates*	*CI*	*p*
Intercept	5.07	4.71 to 5.43	**<0.001**	4.85	4.49 to 5.22	**<0.001**
Negotiate_W_	−0.02	−0.06 to 0.02	0.363	−0.03	−0.08 to 0.02	0.204
Negotiate_B_	−0.16	−0.33 to 0.01	0.058	−0.18	−0.35 to −0.01	0.043
Time	−0.13	−0.17 to −0.09	**<0.001**	−0.00	−0.01 to 0.00	0.306
**Random Effects**						
σ^2^	5.43			4.43		
τ_00_	5.91 _ID_			6.11 _ID_		
ICC	0.52			0.58		
N	200 _ID_			200 _ID_		
Observations	4068			2835		
R^2^	0.016			0.013		

*Note.* W = within-participant, B = between-participant, Significant associations are denoted in bold in the table.

### Qualitative results

**Figure 1.**
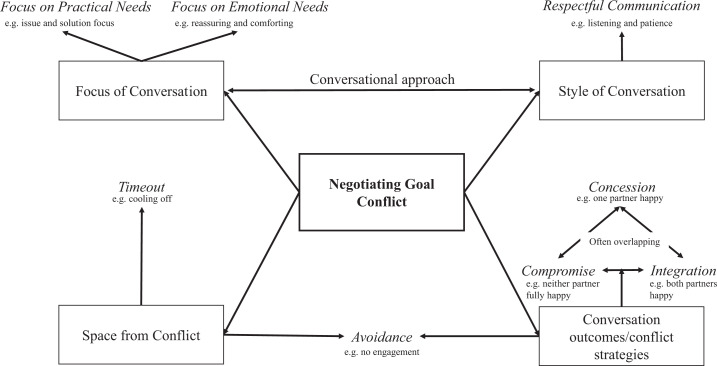
A mind map illustrating how the qualitative themes relate to each other.

The quotes are accompanied with participant number, gender, and age. In the spirit of thematic analysis, no frequencies are reported as these would not be meaningful (Braun & Clarke, 2020). Table 6 presents additional representative quotes. Goal conflict negotiation strategies were divided into six main themes with one of the themes including three subthemes. A mind map illustrating how the different themes related to each other can be found in Figure 1. Overall, most participants described strategies that were helpful but a few also commented on strategies that they had tried in the past and did not find helpful.

**Table 6.** Themes and subthemes with descriptions and representative quotes for negotiation of goal conflict.

Themes	Subthemes	Description	Quotes
Respectful communication		Partners talking honestly, respectfully, and clearly; remaining open to partner’s thoughts; using humor; and not forcing communication.	Phrasing things in such a way that it’s not like a command, for starters. (#17, W, 41) Well, sort of, you know, getting annoyed about people getting too entrenched in their points of view early on, and then it being hard to resolve either way. (#31, M, 29) Continuing to talk about something once we’re upset. So once we get to a point of being too upset in an argument, but continuing to drive the point that we’re trying to make when nobody is listening, really doesn’t help. (#14, W, 30) I never want to seem pushy. Yeah, I’m more likely to kind of stay quiet unless I have a strong opinion on something. (#21, W, 25)
Talk about it	Compromise	An acceptable middle ground is found between both partners ideas	We always managed to find an outcome that we’re both happy with. Whether it’s a compromise or whether we bring [round the other person’s thinking] (#11, W, 36) So talk about it and see, you know, explain our points of view and then see if we can reach a compromise. (#31, M, 29) I think just like being aware of the other person’s perspective. […] So I think that the understanding of the person’s perspective and just trying to be chill about stuff and finding alternative versions. (#32, W, 36)
	Integration	Partners work together to find a solution that is good for them both	We sort of talk through the pros and cons of each thing that both of us wants to do. (#38, M, 33) Try and invite kind of like joint problem solving and shared responsibility. (#18, W, 32) Talking about all the solutions, and then think about for each solution, what is the pros and cons, advantages and disadvantages. (#7, W, 26)
	Concession	One partner accepts their partners ideas to resolve conflict or concession is expected leading to further conflict	You know, trying to sort of insist we do everything that I want to do to get my stuff out the way first doesn’t work either. (#15, W, 36) For the most part, we negotiate pretty well and sort of reasonably, you know, sort of concede the other person’s point. (#23, W, 49) If I want to do something she doesn’t, most of the time, I’ll just say right, fine. (#26, M, 40) He would try to please me more, I guess. (#39, W, 29)
Focus on emotional needs		Partners focus on and consider how the other is feeling and attempting to understand each other	Explicitly acknowledging that, you know, at the end of the day, we just want what’s best for each other because we care for each other a lot. (#5, W, 36) It’s generally a lot of me asking him questions about how he feels, because I think it’s harder for him to, to just say outright. (#8, W, 27) I think that the understanding of the person’s perspective and just trying to be chill about stuff and finding alternative versions. (#32, W, 36)
Focus on practical solutions		Partners focus on a solution for the conflict and how this can practically be achieved	He’s probably more matter of fact about stuff. I guess [he] probably would go to practical advice quicker. (#11, W, 36) I solve conflicts. I just find the solution if it’s good for me or bad for me, I just find a way to solve problems (#34, M, 18) So we just try to stay as positive as possible. And when we do sit down to talk to each other about a problem, we both tend to try to bring solutions to the table. Not only what the problem is. (#36, W, 52)
Timeout		During a heated discussion partners take space away from one another before reconvening	The communication will stop for a little while and we’ll both go off and calm down. And once we’ve both calmed down and then can come back together and say well I was angry or unhappy or stressed out because of XYZ. (#17, W, 41) But there are situations where when we do have an argument and we talk, sometimes it’s not communicating that helps us at times, there has to be space. (#30, W, 39)
Avoidance		Partners avoid conversations of contentious issues. E.g.	I don’t know to be honest, because sometimes it doesn’t feel like we do [reach a compromise or decision]. (#18, W, 32) When the feeling it’s negative, I’m leaving the house going for a walk. (#35, M, 64) He wants to avoid any form of conflict at any point and would probably see this obviously, it’s quite stressful. It’s something you’d probably rather avoid. (#8, W, 27)

#### Respectful communication

Most participants described their negotiation of goal conflict as involving strategies that included clear and respectful communication (as opposed to accusatory or negative communication), flexibility, use of humor, and trying not to force communication. Many participants stated that engaging in “open and honest” (#13, M, 31 and #16, W, 23) communication while “not accusing [partner] of anything” (#2, W, 37) was effective. Furthermore, one participant noted that when they felt hurt, they “would reciprocate with disrespectful words” but typically deemed this to be an unsuccessful strategy in negotiating goal conflict (#37, W, 19). Some participants also noted that flexibility was important when discussing goal conflict. For example, one participant stated that “[It’s] good to listen, good to be flexible, while we’re looking at alternative solutions” (#6, M, 19). Many participants also said that due to the seriousness of the pandemic, using humor was helpful in alleviating any potential conflicts. For example, “Actually, sometimes not being too serious and heavy about it, and just finding I guess the fun. Putting on music and doing silly dances” (#11, W, 36). Additionally, many participants said that pressuring their partner to communicate when they were not ready was not helpful. For example, one participant said “If he is not ready to speak […] I am never ever going to change his perspective, by just badgering him, or throwing information at him or insisting that we talked about it now” (#4, W, 46).

#### Talk about it

Many participants reported that they would discuss any goal conflict to either seek compromise, integrate two partner’s perspectives, or until one person would concede. It was not always clear what the participants’ goals were: whether they wanted to compromise, integrate, or concede. The majority of participants identified *compromise* as a useful strategy to resolve goal conflict. This form of mutual concession led to finding a middle ground between each partners’ ideas. For example, one participant described that they would “kind of focus more on the compromise or some solutions to it that you can both be happy with” (#14, W, 30). Other participants mentioned negotiation leading to *integration* of both partner’s goals in order for both to be happy. Many participants stated pros and cons lists to be useful. For example, one participant said “we would [take] the best parts of both of our ways of dealing with things [to] come up with a solution together” (#44, W, 30). Yet, some participants mentioned that they would just concede to their partner or their partner would concede and found this an effective strategy in resolving goal conflict. For example, one participant retorted “I present an option. If she doesn’t like it I ask her what she wants to change and I’m usually okay with any change” (#34, M, 18). However, a few participants stated that expecting their partner to concede led to further conflict. For example, one participant explained that “just putting my opinion across and expecting it to be taken. Then just waiting until he finally concedes but that’s selfish and it doesn’t work” (#44, W, 30).

#### Focus on emotional needs

Within the goal conflict discussions, some participants reported a greater focus on emotional needs of both partners. For many participants this included “understanding and validating [one partner’s] point” (#7, W, 26) and “giving each other room to speak” (#11, W, 36) as well as checking in during the conversation to ensure they were both comfortable: “sometimes it’s worth checking that he’s actually comfortable with something or that it’s not breeding resentment. I would look for more reassurance than him to see that he’s comfortable with what we’ve agreed” (#13, M, 31). Some participants also recognized that the pandemic is an unusual scenario in which both partners may need to show more patience with another. For example, one participant said “Sort of make allowances for the fact that we spend a lot of time together. It is a different situation. Maybe you do need to be more patient and more compromising than you normally would” (#40, M, 33).

#### Focus on practical needs

Yet, other participants mentioned that focusing on practical solutions was important. For many of these participants this led to a focus on practical issues that could have a solution rather than blaming each other or being inflexible. For example, one participant stated “focusing on what needs to be done to get to a solution rather than like saying, ‘you always want this, or you really want that’” (#14, W, 30) and another said they “try to focus on productive issues” (#3, W, 26).

#### Timeout

Many participants noted that taking a timeout was a helpful strategy instead of trying to discuss potential goal conflicts when upset. As many participants were spending more time together due to the pandemic, many mentioned this was necessary to allow them to cool off. For example, one participant stated “sometimes you have to roll your eyes, go away five minutes, and wait for something and then it’s okay” (#10, M, 42) and another participant said that “if it was gonna get heated or emotional, we both agree to back off the situation for a minute, get some time or some space” (#41, W, 27). Although participants were closer in proximity, this did not mean they were constantly available to discuss potential goal conflicts. Therefore, some participants also said that sometimes it was not the right time to discuss a topic in which case they would agree a time or place to discuss it later. For example, one participant commented that “I just explained that maybe we can have the conversations like an hour later, when I would finish my test. I would be more concentrated on him” (#20, W, 29).

#### Avoidance

A few participants also mentioned they or their partner would avoid discussing goal conflict, however, this was often deemed an unsuccessful strategy. For example, one participant noted that “it might be that we just won’t talk about it” (#33, W, 29) whereas another participant stated that “walking away without comment isn’t helpful” (#11, W, 36).

#### Follow-up interviews

Few changes were identified regarding how participants negotiate conflict a month later. Most participants stated they were trying to engage in clear and respectful communication. This led to behavioral changes such as partners “trying to be more vocal” (#12, W, 26) to ensure emotions did not build overtime. None of the participants noted an increase in conflict, with some mentioning a decrease as they had become “less combative” (#2, W, 37). Overall participants appeared to engage in conversations early on, which prevented the occurrence of a heated conflict. As such negotiating goal conflict became “less confrontational and more conversational” (#12, W, 26).

### Mixed methods results

The mixed methods approach allows for comparison between the quantitative and qualitative results and can be complementary. Both the quantitative and qualitative results indicated relatively low levels of goal conflict during the course of the pandemic. Higher goal conflict was significantly associated with lower goal outcomes in the quantitative data. In the qualitative interviews, most participants could think of at least one scenario during the course of the lockdown that their goals had conflicted and some participants said they would sometimes give up their goals if their partner felt strongly about theirs or they would find an alternative compromise solution. The results from the quantitative analyses also showed that successful negotiation of goal conflict predicted better goal outcomes. Many of the interview participants reported that they were able to negotiate potential goal conflicts relatively seamlessly and many said they would find a mutually satisfying solution in which both partners would be able to pursue their goals.

## Discussion

High goal conflict has been shown to predict negative goal outcomes in previous research (Gere & Impett, 2018). We extend prior work and found that higher goal conflict predicted lower confidence in one’s abilities and lower motivation to pursue goals over days and weeks. Ultimately individuals made less progress toward goals that conflicted with partner’s or relationship’s goals. In addition, in the qualitative interviews, many participants said that if one partner felt their goals were important, the other partner would give up theirs (concession) or adapt their goal in some way (compromise).

Furthermore, the results of the present study also added to the present literature by showing that successful negotiation of goal conflict predicted higher levels of goal outcomes. Our qualitative results shed light into how people negotiate goal conflict in their relationships. We found similar themes to what has been shown in previous quantitative studies which have examined general relationship conflict negotiation (Bonache et al., 2019; Rusbult et al., 1982): compromise, integration, concession, and avoidance. Many participants also said that successful strategies involved both taking each other’s emotional needs into account as well as focusing on workable solutions. Overall, these results suggest that successful negotiation is important in a situation in which one partner’s personal goals conflict with the needs of the relationship or partner.

We also explored whether goal conflict and negotiation of goal conflict were significantly associated with participants’ perception that their goal pursuit was being negatively affected by the pandemic. The results showed that higher goal conflict significantly predicted participants’ perception that the pandemic had negatively affected their ability to pursue goals. In contrast, successful negotiation of goal conflict was unrelated to the perception that the pandemic was affecting goal pursuit. It may be that goal conflict is one way in which participants perceive their goals are being affected by the pandemic. For example, it may be that partners are having to share a tight space with one another and any amount of negotiation cannot completely resolve the problem which means that partners are having to compete for resources to continue to work and pursue other goals.

### Theoretical and practical implications

The present study has several important theoretical and practical implications. Our research shows partners had low goal conflict during the pandemic. These findings are in line with pre-pandemic research that show conflict to be a low frequency experience (Caughlin et al., 2013; McGonagle et al., 1992) and may further suggest key conflicts are of particular importance in the course of a relationship. The study provides further evidence showing that individuals experience a decline in multiple goal outcomes when they experience their goals as conflicting with their partner’s or relationship’s goals. These findings are in accordance with interdependence theory suggesting that goal conflict can be damaging for relationships (Kelley & Thibaut, 1978; Rusbult & Van Lange, 2003). Therefore, in long-term relationships, individuals are likely to devalue conflicting goals for the sake of the relationship (Gere & Impett, 2018). However, the effect sizes in the present study were small with goal conflict only predicting between 2% and 4% of the variance in the outcomes. Successfully negotiating goal conflict may have a stronger positive impact on goal outcomes than goal conflict: negotiation predicted between 6% and 15% of the variance across the outcomes. The findings highlight the importance of negotiating potential goal conflicts so that partners can continue to pursue goals and minimize the impact goal conflict has on goal pursuit. The qualitative findings further our understanding of how partners negotiate instances of context-specific conflict of personal goals. In addition to highlighting different conflict resolution strategies, the findings also suggest that respectfully focusing on both emotional needs as well as practical solutions are needed to successfully negotiate instances of goal conflict. Some participants also highlighted that sometimes taking a timeout before approaching goal conflict was important suggesting that, also in line with previous research (Holley et al., 2013), avoidance, as long as temporary, may be a successful long-term strategy for negotiating goal conflict. These techniques have been noted in previous pre-pandemic research (e.g., Delatorre & Wagner, 2019) which suggests conflict negotiation appears to have been largely unchanged despite the pandemic. Together, both quantitative and qualitative results provide evidence of the importance of negotiating goal conflict in relationships that we would expect to be relevant during and beyond the current global health crisis.

In addition to theoretical implications, the study has several practical implications. The results suggest that successful negotiation of goal conflict is likely to be associated with better outcomes. It may be important to assess the strategies found in the present study in the context of individual goal pursuit and facilitate discussion of goal pursuit and potential goal conflict in couples. Finally, the qualitative results can be used to provide strategies to the public on how to effectively negotiate situations of goal conflict during the pandemic. These may include suggesting taking a timeout before engaging in a conversation about the goal conflict; focusing on both emotional needs and practical solutions; and being clear with each other whether the goal of the negotiation is to integrate, compromise, or concede.

### Strengths, limitations and future directions

The present study had several strengths. We used mixed methods which benefit from the generalizability and reproducibility of quantitative analyses and the nuanced and detailed description of participants’ experiences in the qualitative interviews. The study used longitudinal data with both daily and weekly reports, which enabled us to assess both within- and between-participant change over time. The study had adequate power to estimate hypothesized effects. We used several goal outcome measures to investigate whether (negotiation of) goal conflict was associated with a number of outcomes. All hypotheses, research questions, and analyses were preregistered.

However, the study also had several limitations and the results of the study should be interpreted with these in mind. The data were only collected from individuals in a couple, not dyads, and was therefore based on one partner’s perception. In the interviews, the participants were asked about their own as well as their partner’s behavior, but the participants’ reports of their partner’s behavior may be less accurate. For example, it may be that partners have a different perception of which goals are conflicting. Additionally, in situations in which partners’ goals conflict with each other’s, one member of the couple may end up sacrificing their goals for the other partner’s, which may have implications for relationship and individual well-being. Future research should therefore assess these questions in a sample of dyads.

Overall, the level of goal conflict was also very low in the present sample. The sample was likely to include individuals who were more available and less affected by the pandemic and thus able to participate in the study. As such, the effective strategies noted by the participants may not be representative of those who were highly stressed or had experienced larger changes due to the pandemic. Additionally, 30% of the time the goals that participants were reporting on were domestic and therefore may conflict less on a day-to-day basis. It is likely that many potential high conflict goals such as moving away to study, increasing hours at work, or making a high-risk investment have been put on hold during the pandemic. Therefore, future research should focus specifically on understanding the impact of high conflict goals. Experimental evidence on the impact of goal conflict on goal outcomes is also lacking and future research is needed to investigate these associations in experimental settings. For example, researchers could manipulate goal conflict to examine whether higher levels of goal conflict predict participants’ attitudes toward pursuing the goal.

There are also other limitations due to the nature of the pandemic. The study was only able to capture 5 weeks of lockdown and it is possible that these results would change over time as lockdown measures are eased and people are able to pursue potentially higher conflict goals. Partners’ goals especially related to the pandemic may also conflict. For example, one partner may feel more comfortable with easing of social distancing or flying overseas for a vacation whereas another partner may prefer to act more cautiously. It would be interesting to also understand how couples negotiate how to navigate a need for social contact and connection with a need for health and safety during the pandemic.

## Conclusion

The present mixed methods study provided both quantitative and qualitative evidence on how goal conflict and negotiation of goal conflict is associated with goal outcomes. The results supported the novel preregistered primary hypotheses and were relatively consistent across analyses: higher goal conflict was negatively associated with goal outcomes whereas successful negotiation of goal conflict was positively associated with goal outcomes. The qualitative interviews highlighted several ways in which partners were able to negotiate instances of goal conflict and suggested that over the course of the pandemic, participants became even better at negotiating goal conflict, perhaps because they had more practice with smaller day-to-day conflicts. Overall, most participants reported that the pandemic was affecting their goal pursuit at least somewhat. However, successful negotiation of goal conflict can buffer against potential negative outcomes.

## Notes

1. We did not invite participants who completed the interview after the first 2 weeks to the follow-up interviews as these participants were interviewed for the first time around the time the first participants were having their second interviews.

2. The tables show which variables were in the models in the analyses. We also reran the analyses with gender, age, presence of children, and relationship length included as covariates. None were significant and none changed the results.

3. Because of the way the variables were measured (0–10 for all variables except for 0–100 for goal progress), the effect sizes can be read as they are. For example, for every 10% increase in negotiation of goal conflict, there was a corresponding 2.16% increase in goal progress (Table 4).
